# Ultrastructural and Morphological Effects in T-Lymphoblastic Leukemia CEM-SS Cells Following Treatment with Nordamnacanthal and Damnacanthal from Roots of *Morinda elliptica*

**DOI:** 10.3390/molecules27134136

**Published:** 2022-06-28

**Authors:** Saiful Yazan Latifah, Banulata Gopalsamy, Raha Abdul Rahim, Abdul Manaf Ali, Nordin Haji Lajis

**Affiliations:** 1 Department of Biomedical Sciences, Faculty of Medicine and Health Sciences, Universiti Putra Malaysia, Serdang 43400, Malaysia; banulatagopalsamy@gmail.com; 2 Department of Cell and Molecular Biology, Faculty of Biotechnology and Biomolecular Sciences, Universiti Putra Malaysia, Serdang 43400, Malaysia; raha@upm.edu.my; 3 Faculty of Bioresources and Food Industry, Universiti Sultan Zainal Abidin (UniSZA), Kuala Terengganu 20300, Malaysia; manaf@unisza.edu.my; 4 Laboratory of Natural Products, Institute of Bioscience, Universiti Putra Malaysia, Serdang 43400, Malaysia; nordinlajis@gmail.com

**Keywords:** damnacanthal, nordamnacanthal, CEM-SS, T-lymphoblastic leukemia

## Abstract

Background: *Morinda elliptica* (family Rubiaceae), locally known as ‘mengkudu kecil’, has been used by the Malays for medicinal purposes. Anthraquinones isolated from the roots of *Morinda elliptica*, namely nordamnacanthal and damnacanthal, have been widely reported to exhibit anticancer and antioxidant properties in various cancer models in vitro and in vivo. Aim: This study analyzed the morphological and ultrastructural effects of damnacanthal and nordamnacanthal on T-lymphoblastic leukemia CEM-SS cells. Method: Light microscopy, Giemsa staining, Wright’s staining, scanning electron microscopy, and transmission electron microscopy were carried out to determine apoptosis, necrosis, and ultrastructural changes that occurred within the cells. Results: The outcomes showed that these compounds induced cell death by apoptosis and necrosis, specifically at higher doses of 10 and 30 μg/mL. Condensation and fragmentation of the nuclear chromatin, which further separated into small, membrane-bound vesicles known as apoptotic bodies, were observed in the nuclei and cytoplasm. The plasma membranes and cytoskeletons also showed marked morphological changes upon treatment with damnacanthal and nordamnacanthal, indicating apoptosis. Conclusion: Therefore, we report that damnacanthal and nordamnacanthal exhibit anticancer properties by inducing apoptosis and necrosis in CEM-SS cells, and they have potential as a drug for the treatment of T-lymphoblastic leukemia.

## 1. Introduction

The incidence of cancer has increased dramatically in the last few decades worldwide. Cancer is the second leading cause of death after heart disease in the United States of America [1]. Globocan [2] reported that 19.3 million cancer cases and 10 million cancer-related deaths occurred globally in 2020. The three most common cancer diagnoses are breast (11.7% of all new cases), lung (11.4%), and colorectal cancer (10.0%). The primary forms of cancer mortality are lung cancer, followed by colorectal and liver cancers, accounting for 18.0%, 9.4%, and 8.3% of all cancer deaths, respectively [2]. In Malaysia, a total of 48,639 new cases and 29,530 cancer deaths occurred in 2020 [2].

T-lymphoblastic leukemia is an aggressive form of a hematological tumor. Lymphoid precursor cells, namely lymphoblasts, that undergo cell cycle arrest during early cancer development become the precursor malignant cells for lymphoblastic leukemia [3]. The early cell arrest is triggered by abnormal gene expression due to any abnormalities of chromosomal numbers or chromosomal translocations [3]. Globocan [2] reported the worldwide incidence of general leukemia to be 474,519 cases with a mortality rate of 311,594 in 2020 for both sexes and all ages.

The steadily increasing interest in the development of drugs against cancer particularly started in the 1940s [4]. In general, anticancer drugs stop cancer cell growth or multiplication at one or more points in their life cycle. They interfere with cell replication to cause either tumor cell killing (cytotoxic drugs) or cessation of growth (cytostatic drugs) [5]. Even though chemotherapy can now be considered the main curative treatment, drugs for cancer are not only becoming impersonal but also expensive and highly genotoxic, teratogenic, and fetotoxic [6,7]. In certain cases, drugs may increase the risk of developing a second cancer [8].

Natural products, especially higher plants, have historically served and remain as templates for the development of many important classes of drugs, such as mitotic inhibitors and antibiotics. The World Health Organization (WHO) estimated that at least 80 percent of the world’s population relies mainly, if not totally, on natural medicines. These pharmaceuticals are derived either directly or indirectly from natural resources [9,10]. Phytosterols and phytocomplexes, which are found in different parts of plants, possess profound biological benefits such as anti-inflammatory, antioxidant, and anticancer properties [11,12,13]. The therapeutic properties of plants have been subjected to continuous assessment and evaluation not only recently but over thousands of generations. Through previous efforts, plants have become a viable source of clinically useful anticancer agents, such as vincaleukoblastine (vinblastine; Velban^®^) and leurocristine (vincristine; Oncovin^®^) from Catharanthus roseus [14] and taxol (paclitaxel; Taxol^®^) from Taxus brevifolia [15].

*Morinda elliptica*, locally known as mengkudu kecil (little mengkudu) or mengkudu hutan (woodland mengkudu), is a small wild tree commonly found in open, cleared ground, especially in lalang fields and on rocks by the sea, in a few Malaysian states. As it is very common and almost always available, it is mostly used by the Malays for medicinal purposes. Its very bitter-tasting leaves are eaten with rice as a tonic to restore appetite. Furthermore, it is used to treat fever, leucorrhea, wounds, headaches, cholera, diarrhea, hemorrhoids, and convulsions, and these traditional uses have been documented as early as 1935 [16,17].

**Figure 1:**
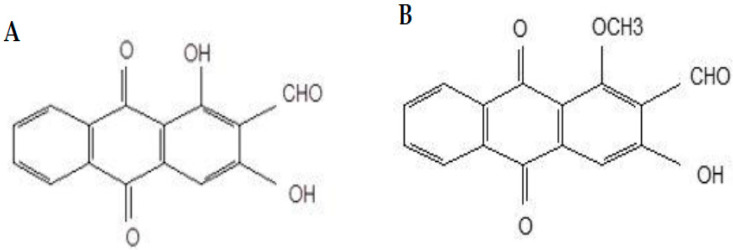
Chemical structures of nordamnacanthal (**A**) and damnacanthal (**B**).

Damnacanthal and nordamnacanthal (Figure 1) are two naturally occurring anthraquinones present in *M. elliptica* that have been reported to possess good anticancer properties. Anthraquinones isolated from Morinda sp. Have exhibited strong antitumor activities and free-radical-scavenging activity, indicating their potent antioxidant properties [18,19,20]. Nordamnacanthal exhibited anticancer and antioxidant activities towards B-lymphoblastoid cell lines [19] and breast cancer MDA-MB231 and inhibited 4T1 tumor progression [21]. Damnacanthal, on the other hand, was reported for its cytotoxic effect towards small-cell lung cancer cell lines [22]. Hiramatsu et al. [23] reported damnacanthal’s anticancer properties towards leukemia, lung, and colon cancers in humans. Both damnacanthal and nordamnacanthal were cytotoxic towards breast carcinoma MCF-7 [24].

Latifah et al. [25] showed that the programmed cell death induced by these anthraquinones was activated by Mg^2+^/Ca^2+^-dependent endonuclease, which led to DNA fragmentation. Nordamnacanthal induced apoptosis, which happened independently from ongoing transcription processes in CEM-SS cells, whereas damnacanthal exhibited a cytostatic effect on human CEM-SS cells by causing cell cycle arrest at the G0/G1 phase. The cytotoxic and the cytostatic effects were, however, not connected.

These anthraquinones induce apoptosis in cancer cells. The morphological hallmarks of apoptosis are irreversible chromatin condensation, fragmentation of DNA, and the compacting of cytoplasm. The plasma membrane forms protrusions, leading to the breaking of the nucleus. The cells then detach from their respective surrounding tissues, forming cell fragments. The cytoplasm of the fragmented cells is compacted with organelles and is termed an apoptotic body. The budding process then occurs when the markers at the cell surface, specifically phosphatidylserine, boost the phagocytosis process with macrophages and parenchyma, which clear the degraded bodies, preventing the occurrence of necrosis [26].

This study reports the effect of damnacanthal and nordamnacanthal (Figure 1) treatment at a cellular level and the ultrastructural changes that happen in T-lymphoblastic leukemia (CEM-SS) cells. We also report the cell viability, apoptosis, and necrosis that occur in CEM-SS cells following treatment with these anthraquinones.

## 2. Results

The cytotoxic concentrations of nordamnacanthal and damnacanthal against several different normal and T-lymphoblastic leukemia cell lines ranged from 1.7 to 30 μg/mL (Table 1). CEM-SS cells were found to be sensitive towards nordamnacanthal, with an IC_50_ value of 1.7 μg/mL. Since the IC_50_ values can have such a range of concentrations, 1, 3, 10, and 30 μg/mL were selected to test the cytotoxicity of these anthraquinones on CEM-SS cells. On the other hand, CEM-SS cells are less sensitive towards damnacanthal, recording an IC_50_ value of 10 μg/mL. Both nordamnacanthal and damnacanthal were less toxic towards normal cell lines, PBMCs, 3T3, and Vero, with an IC_50_ value of more than 30 μg/mL.

**Figure 2:**
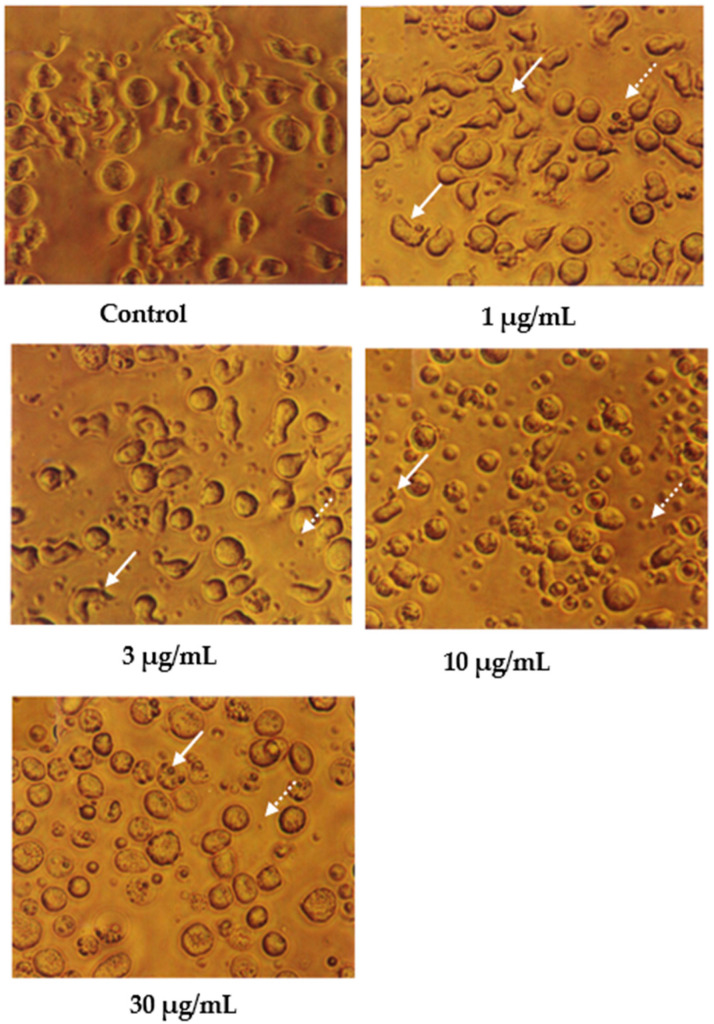
CEM-SS cells without treatment (control) or treated with different concentrations of nordamnacanthal for 24 h (200× magnification). Complete arrows indicate apoptotic cells, and dotted arrows indicate necrotic cells.

Cells undergoing apoptosis for 24 h of treatment were observed under a light microscope, as shown in Figure 2. The control (untreated) cells appeared to be large and healthy, and they came in irregular shapes and sizes. Irregular, hair-like projections from the cytoplasm or the surface microvilli could be mostly seen in every individual cell. In the treatments employing 1 and 3 μg/mL of nordamnacanthal, the existence of apoptotic cells and apoptotic bodies that were randomly distributed in the culture was detectable. The number of these apoptotic cells and bodies increased dramatically for the treatments with higher concentrations of nordamnacanthal (10 and 30 μg/mL).

In the treatment using 30 μg/mL of nordamnacanthal, healthy cells were hardly seen, with almost all the cells showing characteristic changes of apoptosis. The surface microvilli totally disappeared, and the organelles became compacted together. In some cells, cytoplasmic protrusions known as blebs were observed, which eventually gave rise to small cytoplasmic packages called apoptotic bodies. The cells looked smaller in size or shrunken due to a significant reduction in the cell volume. Some cells were in the early stages of apoptosis, with chromatin condensed into the nuclear membrane forming crescent-shaped structures, the easily identifiable morphological features of apoptosis. Some in the later stages showed the collapse of chromatin into two or more spherical masses. The apoptotic bodies appeared to be round or oval masses of cytoplasm and were smaller in size than the cell of origin. Necrotic cells were also observed, even though the number was extremely small compared to the apoptotic cells. These cells seemed swollen, and the nuclei looked normal (Figure 2).

**Figure 3:**
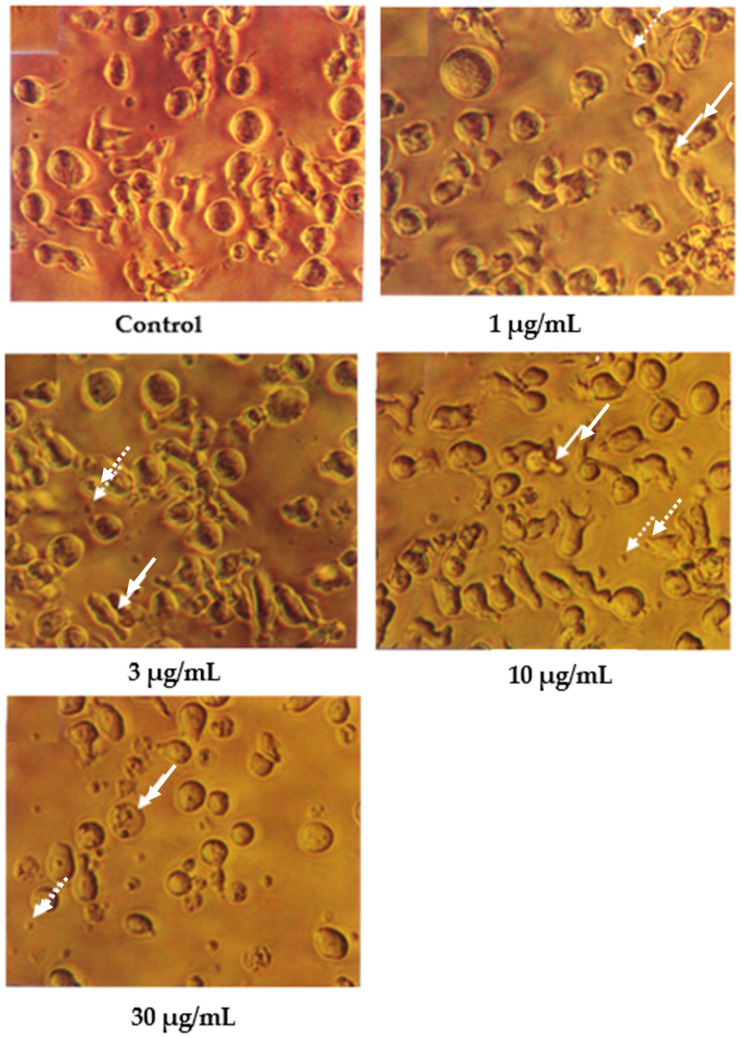
CEM-SS cells without treatment (control) or treated with different concentrations of damnacanthal for 24 h (200× magnification). Complete arrows indicate apoptotic cells, and dotted arrows indicate necrotic cells.

In contrast, the cells treated with 1 and 3 μg/mL of damnacanthal, as shown in Figure 3, showed no morphological differences compared to the untreated cells. The apoptotic cells and apoptotic bodies could be clearly seen in the cells treated with higher concentrations of 10 and 30 μg/mL of damnacanthal. Nevertheless, healthy, unaffected cells could still be observed in this culture.

**Figure 4:**
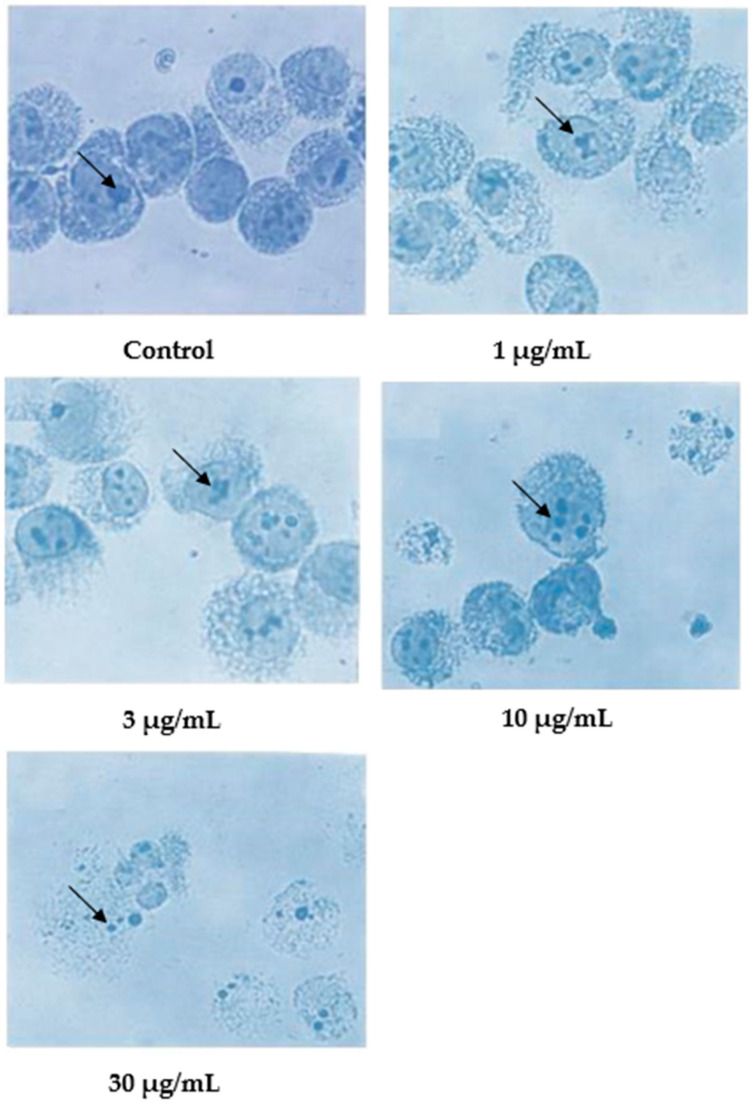
Giemsa-stained CEM-SS cells without treatment (control) or treated with different concentrations of nordamnacanthal for 24 h (200× magnification). Arrows indicate collapsed chromatin.

Following Giemsa staining, as shown in Figure 4, the untreated cells appeared healthy, some were round, and some were irregular in shape and size. The nuclei were large with clear nuclear membranes, and the chromatin was fairly well-distributed in the nuclear compartments. In the treatments using 1 and 3 μg/mL of nordamnacanthal, some cells showed nuclear compaction. Budding and apoptotic cells were noticeable in the cells treated with 10 μg/mL of nordamnacanthal. There were no healthy cells found in the treatment using 30 μg/mL of nordamnacanthal. The chromatin collapsed into two or more spherical masses, and the nuclei seemed to be totally deteriorated (Figure 4).

**Figure 5:**
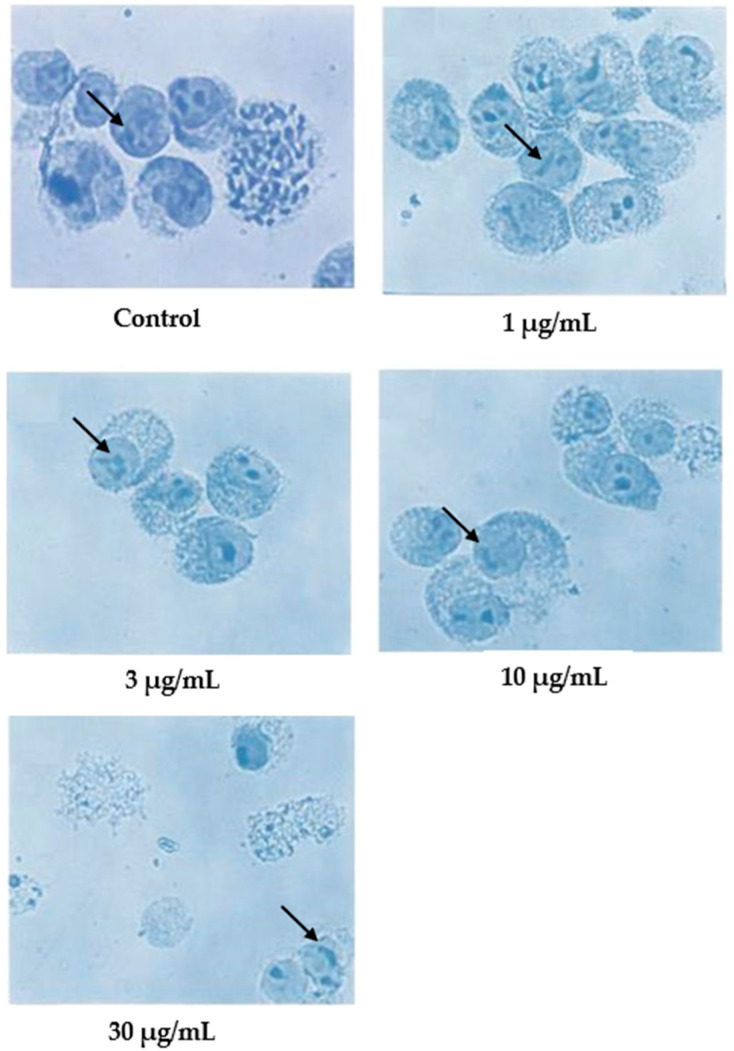
Giemsa-stained CEM-SS cells without treatment (control) or treated with different concentrations of damnacanthal for 24 h (200× magnification). Arrows indicate collapsed chromatin.

In the case of damnacanthal, as shown in Figure 5, nuclear compaction was observable in some cells treated with 10 μg/mL of the compound. Even though most of the cells showed a characteristic morphological pattern of apoptosis (as found in the treatment employing 30 μg/mL of nordamnacanthal), there were still healthy, unaffected cells existing in the culture with 30 μg/mL of the damnacanthal treatment.

**Figure 6:**
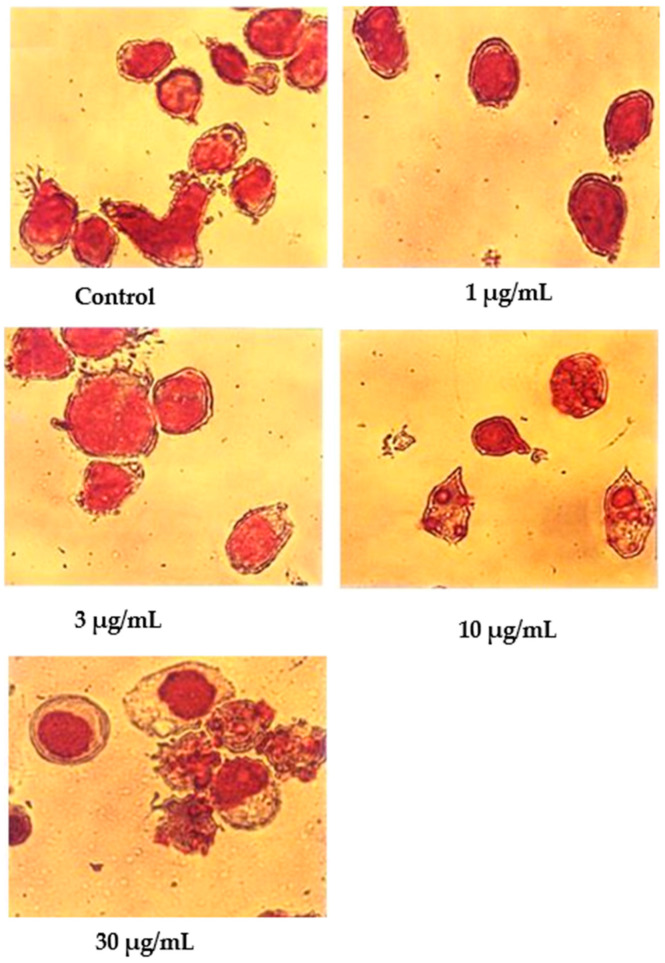
Wright’s-stained CEM-SS cells without treatment (control) or treated with different concentrations of nordamnacanthal for 24 h (200× magnification).

Wright’s stain was used to identify apoptotic cells under light microscope observation after 24 h of treatment with nordamnacanthal (Figure 6). The untreated cells were healthy with large nuclei. Some appeared round, and some displayed irregular shapes and sizes. The cytoplasmic and the nuclear membranes were clearly seen. The nuclear compaction that refers to the early stages of apoptosis was detectable in the cells treated with 1 and 3 µg/mL of nordamnacanthal. Most of the cells in treatments with higher doses of 10 and 30 µg/mL were in the later stages of apoptosis. At this level, the chromatin had already collapsed into two or more spherical masses.

**Figure 7:**
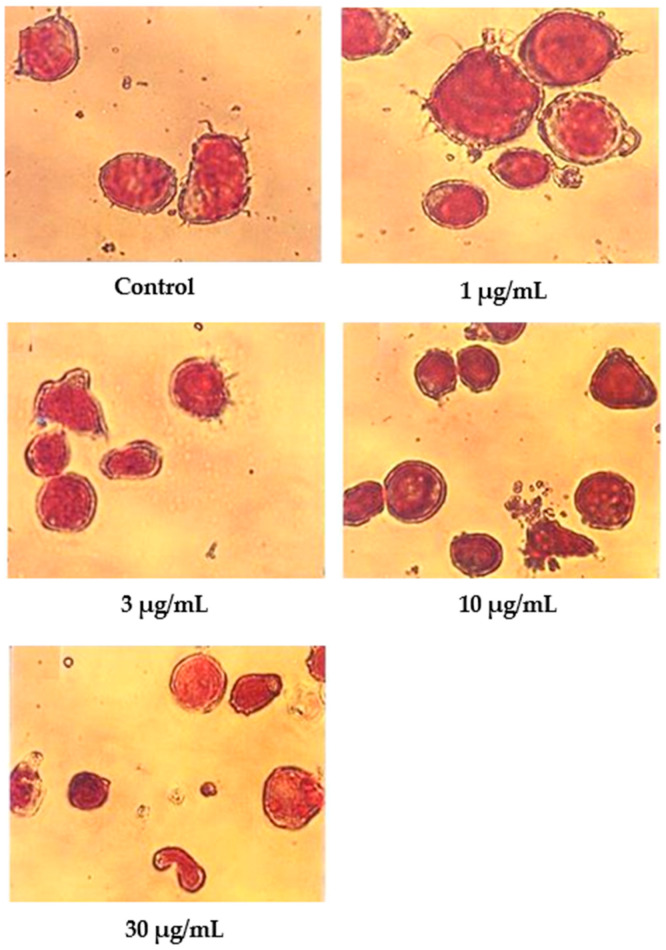
Wright’s-stained CEM-SS cells without treatment (control) or treated with different concentrations of damnacanthal for 24 h (200× magnification).

In contrast to nordamnacanthal, most of the cells treated with 10 μg/mL of damnacanthal were in the early stages of apoptosis (Figure 7). The nuclei were seen to be compacted, and the chromatin condensed into the nuclear membrane, forming crescent-shaped structures. Changes showing the late stages of apoptosis were noticed in the cells treated with 30 μg/mL of this compound. In general, cells that committed apoptosis in the treatments using these two compounds were relatively smaller in size than the healthy ones, and the surface microvilli completely disappeared. On the other hand, there were no marked morphological differences in the cells treated with both 1 and 3 μg/mL of damnacanthal compared to the unaffected cells (Figure 7).

**Figure 8:**
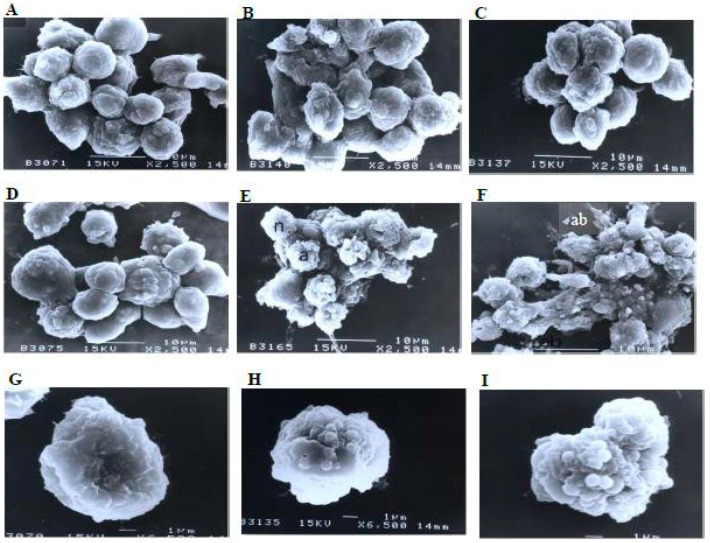
Scanning electron micrographs of CEM-SS cells (**A**) without (control) or treated with (**B**) 1 μg/mL, (**C**) 3 μg/mL, (**D**) 10 μg/mL, or (**E**,**F**) 30 μg/mL of nordamnacanthal for 24 h (2500× magnification). (**G**–**I**) are close-up views of cells treated with 10 μg/mL and 30 μg/mL, respectively, of the compound (6500× magnification). a: apoptotic cell, n: necrotic cell, and ab: apoptotic body.

A scanning electron microscopy analysis (Figure 8) showed that the control (untreated) cells with irregular shapes and sizes appeared healthy. Irregular hair-like projections from the cytoplasm or surface microvilli were mostly seen in every individual cell. Cells treated with 1, 3, and 10 μg/mL of nordamnacanthal showed the earliest stage of apoptosis in the CEM-SS cells. At this point, the microvilli that were originally present disappeared, and blunt protuberances were formed on the cell surface. The focal surface protrusions then became very pronounced in every cell treated with 30 μg/mL of nordamnacanthal. In some cells, the protuberances separated from the cell of origin, producing apoptotic bodies of varying sizes. Nevertheless, necrotic cells were hardly seen, even in the treatment using the highest concentration of nordamnacanthal.

**Figure 9:**
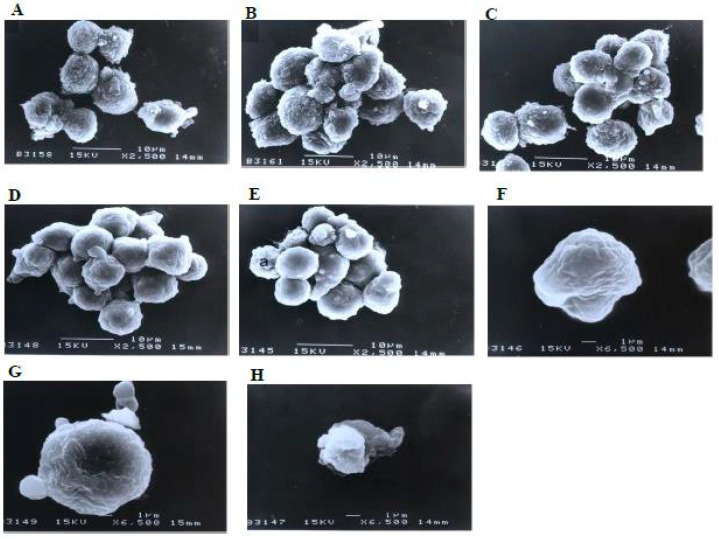
Scanning electron micrographs of CEM-SS cells (**A**) without (control) or treated with (**B**) 1 μg/mL, (**C**) 3 μg/mL, (**D**) 10 μg/mL, or (**E**) 30 μg/mL of damnacanthal for 24 h (2500× magnification). (**F**–**H**) are close-up views of cells treated with 10 μg/mL and 30 μg/mL, respectively, of the compound (6500× magnification). a: apoptotic cell.

Similarly, there were no morphological changes compared to the control in the treatments using 1 and 3 μg/mL of damnacanthal (Figure 9). The microvilli were completely vanished, and blebs started to develop on the surfaces of some cells treated with 10 μg/mL of the compound. The blebs were then completely formed but only in a few cells in the treatment using 30 μg/mL of damnacanthal. The cells undergoing apoptosis shrank and were smaller in size compared to healthy, unaffected ones.

**Figure 10:**
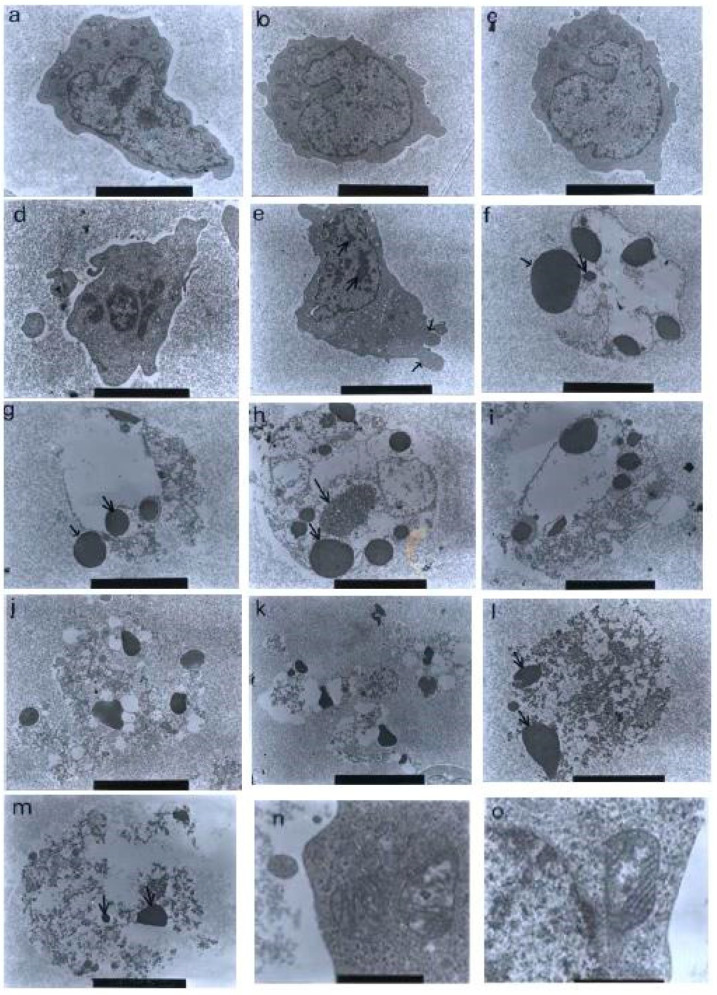
Transmission electron micrographs of CEM-SS cells without treatment (control) or treated with different concentrations of nordamnacanthal for 24 h. (**a**,**b**) Unaffected control cells with irregular shapes. The nuclei were large and chromatin was evenly distributed in the nuclear compartments (6000× magnification). Cells treated with (**c**) 1 μg/mL (7000× magnification) and (**d**) 3 μg/mL (8000× magnification) of nordamnacanthal showing no morphological changes compared to the control. (**e**) Cell treated with 10 μg/mL of nordamnacanthal showing the earliest evidence of apoptosis. The chromatin started to condense (big arrows), and the blebs began to form (small arrows). The surface microvilli totally vanished (5000× magnification). (**f**,**g**) Cells treated with 10 μg/mL with obvious cell and nuclear convolution accompanied by extensive budding, producing distinct nuclear fragments, different in size and chromatin content. The condensed chromatin occupied the whole of the cross-sectional area in some of the fragments (small arrow) and was confined to peripheral crescents in others (big arrow) (7000× magnification). (**h**,**i**) Remnants of the nucleolus were apparent in the nuclei and the nuclear fragments in some planes of the section, appearing in two forms: clusters of dispersed granules (long arrow) and compact granular masses that were closely opposed to the inner surfaces of the condensed chromatin (short arrow) (from 30 μg/mL treatment) (7000× magnification). (**j**) The protuberances on the cell surface began to separate (from 30 μg/mL treatment) (6000× magnification). (**k**) A cluster of apoptotic bodies containing dense and lucent nuclear fragments and well-preserved organelles (from 30 μg/mL treatment) (6000× magnification. (**l**,**m**) Advanced stage of degeneration of apoptotic bodies that could still be recognized by the presence of typical nuclear fragments (arrows) (from 30 μg/mL treatment) (8000× and 10,000× magnification, respectively). (**n**) Mitochondria of control cell (40,000× magnification). (**o**) Unaffected mitochondrion from cell treated with 30 μg/mL of nordamnacanthal (40,000× magnification).

Using a transmission electron microscopy technique, the control (untreated) cells were viewed to have different, irregular shapes and sizes, as shown in Figure 10. The microvilli, in the form of irregular hair-like structures, seemed to be projected from the cytoplasm. The nuclei were large and confined by clear nuclear membranes. The chromatin was evenly distributed in the nuclear compartments. The same observations were also found in the treatments with 1 and 3 μg/mL of nordamnacanthal, showing that the compound at low concentrations did not affect the cells.

The earliest morphological evidence of apoptosis was noticeable in the cells treated with 10 μg/mL of nordamnacanthal. The chromatin began to condense, microvilli that were originally present disappeared, and blunt protuberances started to develop. In some cells, cell and nuclear convolutions were obviously seen. These events were accompanied by more extensive budding, producing discrete nuclear fragments of varying sizes and chromatin contents, which remained surrounded by double membranes (Figure 10).

**Figure 11:**
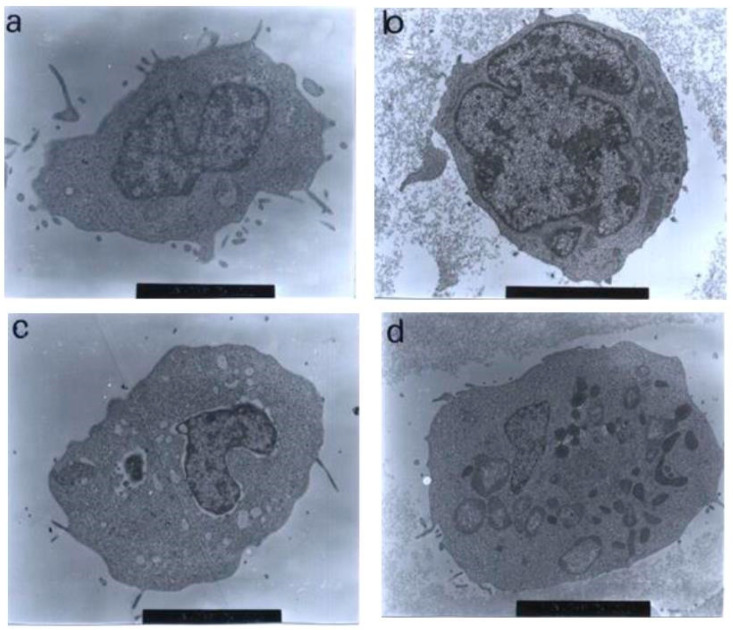
Transmission electron micrographs of CEM-SS cells treated with different concentrations of damnacanthal for 24 h. Cells treated with (**a**) 1 μg/mL (7000× magnification), (**b**) 3 μg/mL (7000× magnification), (**c**) 10 μg/mL (5000× magnification), and (**d**) 30 μg/mL (6000× magnification) of damnacanthal showed no morphological changes compared to the unaffected control cell. The nuclei were large, and the chromatin was well-distributed in the nuclear compartments. Irregular, hair-like projections from the cytoplasm or the surface microvilli could be mostly seen in every individual cell.

In the treatment with 30 μg/mL of nordamnacanthal, almost all the cells were at the stage of extreme cell and nuclear convolutions. The condensed chromatin occupied the whole of the cross-sectional area in some of the fragments, while confined to peripheral crescents in others. Remnants of the nucleolus were apparent in the nuclei and the nuclear fragments in some planes of the sections, and they took two forms: clusters of dispersed granules and compact granular masses that were closely opposed to the inner surfaces of the condensed chromatin. In some cells, the protuberances that formed on the cell surfaces began to separate, producing clusters of membrane-bound apoptotic bodies of roughly spherical or ovoid shapes of varying sizes and compositions. Even though some of the apoptotic bodies were at an advanced stage of degradation, resembling those observed at advanced stages of necrosis, they could still be recognized by the presence of typical nuclear fragments. The structural integrity of organelles such as the mitochondria, on the other hand, remained preserved and unaffected during the process of apoptosis (Figure 10). In the case of damnacanthal, there were no differences between the treated cells, even at the highest concentration (30 μg/mL) of the compound, as compared to the control (Figure 11).

**Figure 12:**
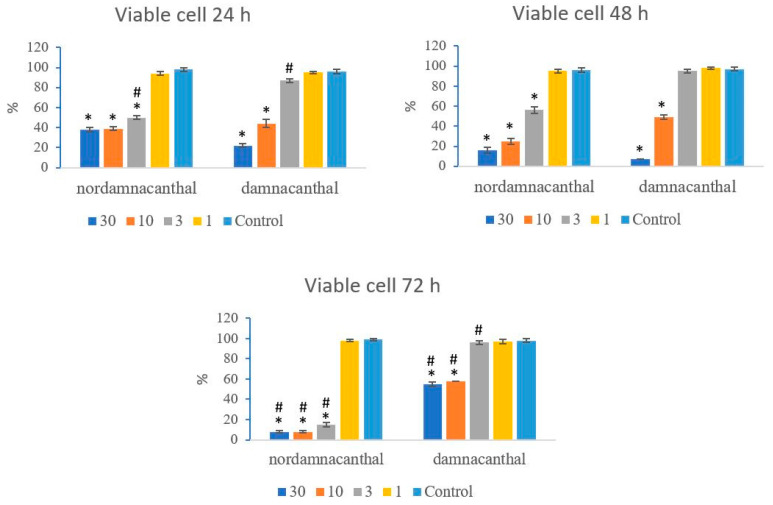
The percentages of viable CEM-SS cells treated with different concentrations of nordamnacanthal and damnacanthal at 24, 48, and 72 h. Control cultures were not treated with nordamnacanthal or damnacanthal. Each data point represents the mean ± SD. * Significant difference of *p* < 0.05 within the same anthraquinone group vs. its respective control. # Significant difference of *p* < 0.05 between nordamnacanthal and damnacanthal of the same concentration.

**Figure 13:**
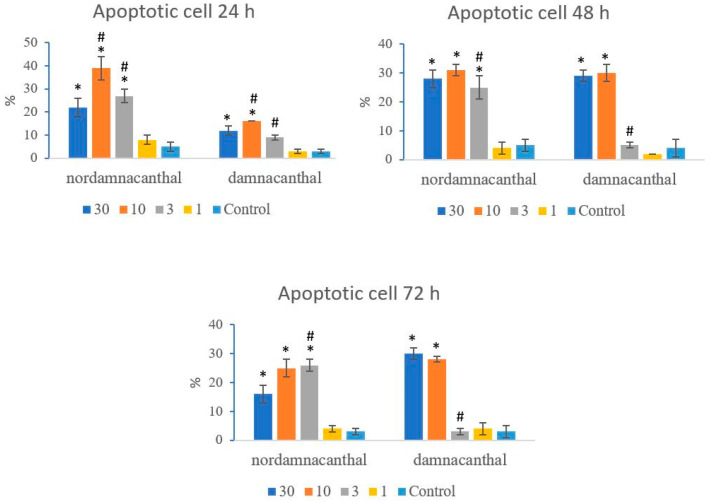
The percentages of apoptotic CEM-SS cells treated with different concentrations of nordamnacanthal and damnacanthal at 24, 48, and 72 h. Control cultures were not treated with nordamnacanthal or damnacanthal. Each data point represents the mean ± SD. * Significant difference of *p* < 0.05 within the same anthraquinone group vs. its respective control. # Significant difference of *p* < 0.05 between nordamnacanthal and damnacanthal of the same concentration.

**Figure 14:**
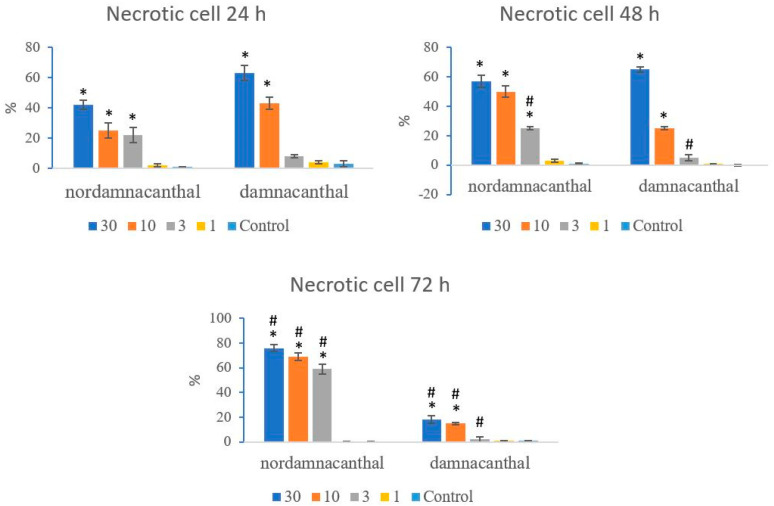
The percentages of necrotic CEM-SS cells treated with different concentrations of nordamnacanthal and damnacanthal at 24, 48, and 72 h. Control cultures were not treated with nordamnacanthal or damnacanthal. Each data point represents the mean ± SD. * Significant difference of *p* < 0.05 within the same anthraquinone group vs. its respective control. # Significant difference of *p* < 0.05 between nordamnacanthal and damnacanthal of the same concentration.

Based on these morphological changes, the numbers of viable, apoptotic, and necrotic cells were counted and expressed as proportions of the total cell number (%). The percentages of viable, apoptotic, and necrotic cells fluctuated throughout the experiment employing both compounds. As shown in Figure 12, following treatment with nordamnacanthal, the percentages of viable cells were lower in the 30, 10, and 3 μg/mL groups compared to control. At 72 h, there was a marked drop in the percentage of viable cells. Treatment with damnacanthal, however, showed a reduction in the number of viable cells, but only in the 30 and 10 μg/mL treatment groups. Figure 13 shows that the percentages of apoptotic cells were also the highest in the 30, 10, and 3 μg/mL treatments of nordamnacanthal. Damnacanthal, on the other hand, only showed high percentages of apoptotic cell for the two highest concentrations (30 and 10 μg/mL). The percentage of necrotic cell in the treatment with 30 μg/mL of nordamnacanthal increased to 57% at 48 h and to 76% at 72 h (Figure 14). The treatment with 1 μg/mL of nordamnacanthal showed an increase in the percentage of viable cells and, thus, gave the lowest proportions of apoptotic and necrotic cells throughout the experiment. The percentage of necrotic cells was increased at 48 h (63%) and reduced at 72 h (18%) following damnacanthal treatment. In two treatments (3 and 1 μg/mL of damnacanthal), the percentages of viable cells were considerably high, and there were no significant differences in the percentages of apoptotic and necrotic cells compared to the untreated culture. Comparatively, nordamnacanthal was more toxic than damnacanthal due to the higher percentage of cell death achieved in the experiment in every concentration used.

## 3. Discussion

The high cytotoxicity exhibited by nordamnacanthal and damnacanthal towards CEM-SS cells while not exerting a toxic effect on noncancerous cells indicated they are potential anticancer agents. At 24 h, nordamnacanthal and damnacanthal caused morphological changes in CEM-SS cells, indicating apoptosis. The cells undergoing apoptosis showed a set of coordinated structural changes that, in the first stage, were characterized by condensation and fragmentation of the nuclear chromatin. During the second stage of the process, there was fragmentation of both the cytoplasm and nucleus, and the cells separated into small, membrane-bound vesicles known as apoptotic bodies [27,28,29]. In addition to the nucleus and cytoplasm, the plasma membranes and cytoskeletons were also shown to undergo marked morphological changes during apoptosis [30,31]. Furthermore, the disappearance of microvilli has been recognized as one of the early events of apoptosis [32,33].

Apoptosis is generally defined as the suicide of a cell established in advance whereby the cell self-destructs to ensure the smooth continuity of normal body functions. Necrosis, on the other hand, is known as untimely cell death that happens as a consequence of extreme and uncontrollable external factors. The factor that causes cell death by apoptosis or necrosis depends on the type of tissue, the stage of development of the tissue, and the death signal, as well as the physiological environment [34,35]. However, in an event where apoptotic cells are not effectively removed by the phagocytic cells, apoptosis can progress into secondary necrosis. In this study, there was no correlation or any dose-dependent relationship between apoptosis and necrosis.

Damnacanthal from *Morinda citrifolia* was previously reported to increase intracellular Ca^2+^ (cytosolic Ca^2+^) by releasing Ca^2+^ from internal stores and promoting Ca^2+^ entry, which may be the possible mechanism that induced apoptosis during testing on human dermal fibroblasts. The damnacanthal-induced Ca^2+^ was found to be mediated by voltage-dependent and voltage-independent Ca^2+^ channels [36]. In fact, it has been shown that the early signal for the initiation of apoptosis by various stimuli was due to elevation in cytosolic Ca^2+^ [30,37]. Intracellular Ca^2+^ levels modulate a multitude of vital cellular processes, including gene expression, cell viability, cell proliferation, cell motility, cell shape, and cell volume regulation, thereby playing a key role in regulating cell responses to external signals. Therefore, the maintenance of low cytosolic Ca^2+^ is necessary for proper functioning of cells [38]. Mitochondria play a vital role in controlling cellular Ca^2+^ dynamics. Mitochondria transport Ca^2+^ to regulate cytosolic Ca^2+^, to serve as a store of Ca^2+^ when its concentration in the cytosol is excessive, to serve as a releasable source of activator Ca^2+^, and to regulate mitochondrial matrix Ca^2+^ and, thereby, control the level of activation of Ca^2+^-sensitive metabolic enzymes. However, we previously reported that elevations in the cytosolic Ca^2+^ concentration were not induced by nordamnacanthal and damnacanthal and were not needed for the apoptosis of CEM-SS cells [25].

Morphological and functional changes in the mitochondria can be caused by oxidative stress after exposure to various toxins that affect both mitochondrial matrix enzymes and membrane-bound enzymes. As a consequence, cytosolic Ca^2+^ increases [39]. Uncontrolled, prolonged elevations of cytosolic Ca^2+^ can result in cell death (apoptosis) because calcium damages cellular proteins and membranes by activating Ca^2+^-dependent degradative enzymes, such as proteases and endonucleases [40], and by promoting free radical production via the activation of lipases [30,41].

In summary, the majority of caspase substrates that have been identified are involved in cell regulation, DNA repair, homeostasis, and cell survival. Cleavage and the subsequent inactivation or alteration of these proteins is a critical event contributing to the apoptotic phenotype [42]. Therefore, the inhibition of CEM-SS cell proliferation by nordamnacanthal and damnacanthal from *Morinda elliptica* seems to be related to their ability to induce apoptosis in the cells.

The occurrence of apoptosis through nordamnacanthal and damnacanthal from *M. elliptica* was found to be extremely concentration- and time-dependent, but the mechanism by which they caused cell death was unknown. Currently available commercial drugs such tamoxifen [43,44], doxorubicin [45], and cisplatin [46] also induce apoptosis to specific cancer cells. Therefore, we report here that nordamnacanthal and damnacanthal induced apoptosis cell death to CEM-SS cells, but the downstream mechanism that leads to this needs to be explored further.

## 4. Materials and Methods

### 4.1. Materials

#### 4.1.1. Cells and Compounds

T-lymphoblastic leukemia CEM-SS cell line and noncancerous cell lines (NIH 3T3: mouse embryo fibroblast; Vero: African monkey kidney fibroblast) with both anchorage-dependent and suspension cells were obtained from the American Type Culture Collection (ATCC), the National Cancer Institute (NCI), and the RIKEN Cell Bank (RCB). Human peripheral blood mononuclear cells (PBMCs) were kindly supplied by Miss Leong Pooi Pooi from the Faculty of Medicine and Health Sciences, Universiti Putra, Malaysia.

#### 4.1.2. Compounds

The anthraquinones, nordamnacanthal and damnacanthal, that were isolated from the roots of *Morinda elliptica*, as outlined in [25,47], were kindly supplied by Prof. Dr. Nordin Haji Lajis from the Department of Chemistry, Faculty of Science and Environmental Studies, Universiti Putra, Malaysia. Briefly, roots of *Morinda elliptica* were collected from Port Dickson, Negeri Sembilan, and identified by Mr Anthonysamy Sivarimuthu from the Department of Biology. A voucher specimen (voucher number: SK2391/14) was deposited in the herbarium of the Department of Biology, Universiti Putra, Malaysia. The roots were air-dried, chopped, ground, and soaked in dichloromethane (CH_2_Cl_2_) at room temperature for 36 h. The solvent was filtered out, and fresh CH_2_Cl_2_ was added. This process was repeated three times. The combined filtrate was evaporated under reduced pressure to give a brown-colored residue. Exactly 12 kg of powdered *M. elliptica* root provided 142 g of residue. Then, 26 g of the crude extract was dissolved in CHCl_3_ and absorbed as a packed 10–50 cm column onto an acid-washed silica gel that was previously shaken for 30 min with 4% oxalic acid, filtered, and dried. Then, based on the TLC patterns, 95 fractions were collected and combined into six major quinone-containing fractions and were labeled A, B, C, D, E, and F for further separation procedures.

After column chromatography, fraction A was re-chromatographed using a smaller 300–350 mm column, packed with 2% acid-washed silica gel, and eluted with a mixture of CH_2_Cl_2_ and petrol. The major component of fractions B and C, separated by TLC in CHCl_3_, was nordamnacanthal. Exactly 1.02 g of nordamnacanthal was isolated after the recovery of the major orange band. The yellow band that appeared after the major orange band of fraction B was then separated using chromatotron eluted with CHCl_3_ to produce 196.4 g of damnacanthal. Absorption spectra were recorded between 190 and 1100 nm. The spectroscopic values of nordamnacanthal and damnacanthal were verified with the literature values [48,49]. The excitation and emission values for nordamnacanthal were 419 nm and 479 nm, respectively, while damnacanthal recorded 248 nm and 314 nm, respectively. The melting point of nordamnacanthal was 214–218 °C, and damnacanthal was 208–211 °C.

The powdered-form compounds (nordamnacanthal and damnacanthal) were dissolved in dimethylsulphoxide (DMSO, Sigma, St. Louis, MO, USA), whereby the percentage of DMSO in the experiment was ensured to not exceed 5%. The solution was prepared with serum-free culture medium (RPMI 1640) and stored at 4 °C.

### 4.2. Methods

#### 4.2.1. Cell Lines

CEM-SS cells were grown in RPMI 1640 medium (Sigma, St. Louis, MO, USA) supplemented with 10% fetal calf serum (Sera Lab, London, UK) and antibiotics (100 units/mL penicillin and 100 µg/mL streptomycin) (Sigma, St. Louis, MO, USA). They were incubated at 37 °C under 5% CO_2_ in a humidified atmosphere. The cells were exponentially growing before all the experiments.

#### 4.2.2. 3-[4,5-dimethylthizol-2-yl]-2,5-diphenyltetrazolium Bromide (MTT) Assay

The cytotoxicity was quantitatively estimated with a nonradioactive colorimetric assay system using tetrazolium salt (MTT, Sigma, St. Louis, MO, USA). Briefly, MTT was dissolved in phosphate-buffered saline at 5 mg/mL and filter-sterilized to remove the small amount of insoluble residue present in some batches of MTT. The MTT stock solution was added directly to all the appropriate microtiter-plate wells (10 µL per 100 µL medium) containing cells and complete growth medium, with or without the tested compounds. The plate was then incubated for 4 h at 37 °C to allow MTT metabolism to formazan. Subsequently, the supernatant was aspirated, and 100 µL of acid with isopropanol (0.04 M HCl in propan-2-ol) was added and mixed thoroughly to dissolve the dark blue formazan crystals. The optical density (OD) was measured with an automated spectrophotometric EL 340 multiplate and microelisa reader (Bio-Tek Instruments Inc., Winooski, VT, USA) using test and reference wavelengths of 570 and 630 nm, respectively. The cytotoxic dose that killed the cells by 50% (IC_50_) was determined from the absorbance (OD) versus concentration curve.

#### 4.2.3. Trypan Blue Dye Exclusion Method

The treatments were carried out in 60 mm petri dishes (Nunc) with a working volume of 5 mL. Briefly, cells with a density of 1 × 10^5^ cells/mL were treated with 30, 10, 3, and 1 µg/mL of nordamnacanthal and damnacanthal. Controls that contained only the cells were included for each sample. The cells were then harvested every 8 h for 3 days (72 h), and the viability was measured by staining the cells with 0.25% trypan blue (Sigma, St. Louis, MO, USA) at a dilution of 1:1 and counting with a Neubauer hemocytometer under a light microscope. The dead cells were stained blue, whereas the viable cells remained clear. The proliferative profiles were determined by comparing the amounts of the treated populations to that of the control cell population.

#### 4.2.4. Giemsa Staining

Briefly, cells with a density of 1 × 10^5^ cells/mL were treated with 30, 10, 3, and 1 µg/mL of nordamnacanthal, damnacanthal, and commercial drugs and were incubated for 24 h. Controls that contained only the cells were included for each sample. The cells were harvested; resuspended in 0.04 M KCl with 0.025 M sodium citrate before adding fresh, ice-cold, glacial acetic acid: methanol (1:3); and spun down at 1000 rpm (200× *g*) for 10 min. This was carried out twice. The supernatant was drained, and the pellet was resuspended in the same ice-cold, glacial acetic acid: methanol solution. Then, one drop of the mixture was placed onto a cold glass slide, dispersed, and air-dried. The slide was then immersed in the Giemsa stain (1 part Giemsa: 9 parts PBS) (Sigma, St. Louis, MO, USA) for 8 min and then washed under running tap water. The slide was visualized under a light microscope.

#### 4.2.5. Wright’s Staining

The staining was carried out as described by Lillie [50]. The treatments were carried out in 60 mm petri dishes with a working volume of 5 mL. Briefly, cells with a density of 1 × 10^5^ cells/mL were treated with 30, 10, 3, and 1 µg/mL of nordamnacanthal and damnacanthal and incubated for 24 h. Controls that contained only the cells were included for each sample. The cells were collected through centrifugation at 1000 rpm (200× *g*) for 10 min. The supernatant was poured out, and the pellet was resuspended in fetal calf serum (Sera Lab, London, UK). One drop of the solution was placed onto a glass slide, dispersed, and air-dried. The slide was flooded with methanol for 5 min, followed by Wright’s stain (Sigma, St. Louis, MO, USA) for 3 min, and subsequently, phosphate-buffered saline was added without washing off the stain. The slide was left at room temperature for 6 to 8 min. The slide was then washed under running tap water and examined under a light microscope.

#### 4.2.6. Scanning Electron Microscopy

Briefly, cells with a density of 1 × 10^5^ cells/mL were treated with 30, 10, 3, and 1 µg/mL of nordamnacanthal and damnacanthal and incubated for 24 h. Controls that contained only the cells were included for each sample. The cells were harvested, washed with PBS, and fixed with 4% buffered glutaraldehyde for 12 to 24 h at 4 °C. They were then washed with 0.1 M sodium cacodylate buffer for 3 changes of 10 min each, post-fixed in 0.5 mL 1% buffered osmium tetroxide at 4 °C for 1 h, and washed again with 0.1 M sodium cacodylate. The cells were then dehydrated in a series of 35% to 50% to 75% to 95% of acetone for 5 min each and, finally, with 3 changes of 100% acetone for 10 min each. The cells were then placed onto albumin-coated aluminum foil, transferred into a specimen basket, and dried with a critical-point dryer (Hitachi HCP-2 Critical Point Dryer, Tokyo, Japan) for about 30 min. The foil was coated with a thin layer of cold in a sputter coater (Polaron E5100, SEM Coating Unit, Hertfordshire, UK). The cells were examined with a scanning electron microscope (JEOL JSM-6400, Scanning Microscope, Tokyo, Japan).

#### 4.2.7. Transmission Electron Microscopy

Cells with a density of 1 × 10^5^ cells/mL were washed with sterile, distilled water and spun down at 1000 rpm (200× *g*) for 10 min. The supernatant was poured out, and the pellet was fixed in 4% buffered glutaraldehyde overnight. The buffer was discarded, and a drop of serum was put into a tube. Glutaraldehyde was added and fixed for 4 h. The pellet was then taken out, cut into pieces, and transferred to a new vial. The pieces were washed with 0.1 M sodium cacodylate buffer for 3 changes of 10 min each, post-fixed in 0.5 mL 1% buffered osmium tetroxide at 4 °C for 1 h, and washed again with 0.1 M sodium cacodylate. The pieces were dehydrated in increasing concentrations of alcohol from 50% (15 min), to 50% (15 min), to 75% (15 min), to 95% (15 min, twice), to 100% (30 min, twice) and were finally cleared in 100% acetone (10 min, twice). Infiltration was carried out in an acetone and pure epoxy resin mixture (1: 1, 2 h; 1: 3, 2 h; 100% resin, overnight; 100% resin, 2 h). The pieces were placed into beam capsules and embedded in epoxy resin. Polymerization was conducted in an oven at 60 °C for 24 to 48 h. Then, 1 µm thick sections were stained with toluidine blue, washed, and examined under a light microscope.

Later, selected areas of interest were cut for either silver or golden ultrathin sections. They were picked up with a grid and dried using filter paper. The sections were then stained with uranyl acetate for 10 min and washed with 50% filtered alcohol. Finally, they were stained with lead citrate for 10 min, washed with double-distilled water, and examined with a transmission electron microscope (Hitachi H-70100 Transmission Electron Microscope, Tokyo, Japan).

### 4.3. Data Analysis

The data were presented as mean ± SEM. The statistical differences between the treated and control groups were determined using Student’s *t*-test. One-way analysis of variance (ANOVA) was also used for multiple comparisons, where *p* < 0.05 was considered to be statistically significant.

## 5. Conclusions

Nordamnacanthal and damnacanthal from the roots of *Morinda elliptica* imparted a cell growth inhibitory response in CEM-SS cells via the induction of apoptosis and necrosis. Nordamnacanthal and damnacanthal caused condensation and fragmentation of the nuclear chromatin, which then led to the fragmentation of cytoplasm and nuclei, forming apoptotic bodies. Furthermore, marked morphological changes to the plasma membrane and cytoskeleton, as well as the disappearance of microvilli, provided evidence of their apoptotic effects on DEM-SS cells.

The purposes of identification and development of new treatments are focal points in translational research on leukemia, a common type of cancer in children. Nordamnacanthal and damnacanthal could possibly be candidates that meet the demands for new, potent anti-acute lymphoblastic leukemia drugs.

## Figures and Tables

**Table 1:** Cytotoxicity of nordamnacanthal and damnacanthal against different cell lines based on IC_50_ values determined by the MTT assay.

Cell Line	Nordamnacanthal	IC_50_ (μg/mL)	Damnacanthal
T-lymphoblastic leukemia (CEM-SS)	1.7 ± 0.4		10 ± 0.5
Human peripheral blood mononuclear cells (PBMCs)	>30		>30
Mouse embryo (3T3)	>30		>30
Monkey kidney fibroblast (Vero)	>30		>30

## Data Availability

Data are contained within the article.

## Sample Availability

Samples of the damnacanthal and nordamnacanthal compounds are available from the authors.

## References

[1] CDC (2017). Deaths and Mortality. https://www.cdc.gov/nchs/fastats/deaths.htm.

[2] GLOBOCAN (2020). New Global Cancer Data. https://www.uicc.org/news/globocan-2020-new-global-cancer-data.

[3] Fattizzo B., Rosa J., Giannotta J.A., Baldini L., Fracchiolla N.S. (2020). The Physiopathology of T- Cell Acute Lymphoblastic Leukemia: Focus on Molecular Aspects. Front. Oncol..

[4] Quirke V. (2014). Targeting the American market for medicines, ca. 1950s–1970s: ICI and Rhône-Poulenc compared. Bull. Hist. Med..

[5] Anttila J.V., Shubin M., Cairns J., Borse F., Guo Q., Mononen T., Vázquez-García I., Pulkkinen O., Mustonen V. (2019). Contrasting the impact of cytotoxic and cytostatic drug therapies on tumour progression. PLoS Comput. Biol..

[6] Ponticelli C., Moroni G. (2018). Fetal Toxicity of Immunosuppressive Drugs in Pregnancy. J. Clin. Med..

[7] Swift L.H., Golsteyn R.M. (2014). Genotoxic anti-cancer agents and their relationship to DNA damage, mitosis, and checkpoint adaptation in proliferating cancer cells. Int. J. Mol. Sci..

[8] Demoor-Goldschmidt C., de Vathaire F. (2019). Review of risk factors of secondary cancers among cancer survivors. Br. J. Radiol. Suppl..

[9] Newman D.J., Cragg G.M. (2016). Natural Products as Sources of New Drugs from 1981 to 2014. J. Nat. Prod..

[10] Veeresham C. (2012). Natural products derived from plants as a source of drugs. J. Adv. Pharm. Technol. Res..

[11] Gupta A.K., Dhua S., Sahu P.P., Abate G., Mishra P., Mastinu A. (2021). Variation in Phytochemical, Antioxidant and Volatile Composition of Pomelo Fruit (*Citrus grandis* (L.) Osbeck) during Seasonal Growth and Development. Plants.

[12] Mastinu A., Bonini S.A., Premoli M., Maccarinelli G., Mac Sweeney E., Zhang L., Lucini L., Memo M. (2021). Protective Effects of Gynostemma pentaphyllum (var. Ginpent) against Lipopolysaccharide-Induced Inflammation and Motor Alteration in Mice. Molecules.

[13] Abate G., Zhang L., Pucci M., Morbini G., Mac Sweeney E., Maccarinelli G., Ribaudo G., Gianoncelli A., Uberti D., Memo M. (2021). Phytochemical Analysis and Anti-Inflammatory Activity of Different Ethanolic Phyto-Extracts of *Artemisia annua* L.. Biomolecules.

[14] Neuss N., Neuss M.N., Brossi A., Suffness M. (1990). Chapter 6 Therapeutic Use of Bisindole Alkaloids from Catharanthus. The Alkaloids: Chemistry and Pharmacology.

[15] Wani M.C., Taylor H.L., Wall M.E., Coggon P., McPhail A.T. (1971). Plant antitumor agents. VI. Isolation and structure of taxol, a novel antileukemic and antitumor agent from Taxus brevifolia. J. Am. Chem. Soc..

[16] Gimlette J.D. (1971). A Dictionary of Malayan Medicine.

[17] Abu Bakar F.I., Abu Bakar M.F., Rahmat A., Abdullah N., Sabran S.F., Endrini S. (2018). Anti-gout Potential of Malaysian Medicinal Plants. Front. Pharmacol..

[18] Ismail N., Mohamad H., Mohidin A., Lajis N.H. (2002). Antioxidant activity of anthraquinones from Morinda elliptica. Nat. Prod. Sci..

[19] Jasril, Lajis N.H., Mooi L.Y., Abdullah M.A., Sukari M.A., Ali A.M. (2003). Antitumor promoting and actioxidant activities of anthraquinones isolated from the cell suspension culture of Morinda elliptica. Asia-Pac. J. Mol. Biol. Biotechnol..

[20] Shami A.M.M. (2018). Antibacterial and antioxidant properties of anthraquinones fractions from Morinda Citrifolia fruit. J. Rep. Pharm. Sci..

[21] Abu N., Zamberi N.R., Yeap S.K., Nordin N., Mohamad N.E., Romli M.F., Rasol N.E., Subramani T., Ismail N.H., Alitheen N.B. (2018). Subchronic toxicity, immunoregulation and anti-breast tumor effect of Nordamnacantal, an anthraquinone extracted from the stems of *Morinda citrifolia* L.. BMC Complement Altern Med..

[22] Kanokmedhakul K., Kanokmedhakul S., Phatchana R. (2005). Biological activity of Anthraquinones and Triterpenoids from Prismatomeris fragrans. J. Ethnopharmacol..

[23] Hiramatsu T., Imoto M., Koyano T., Umezawa K. (1993). Induction of normal phenotypes in ras-transformed cells by damnacanthal from Morinda citrifolia. Cancer Lett..

[24] Ali A., Ismail N., Mackeen M., Yazan L.S., Mohamed S.M., Ho A.S., Lajis N.H. (2000). Antiviral, cyototoxic and antimicrobial activities of anthraquinones isolated from the roots of Morinda elliptica. Pharm. Biol..

[25] Latifah S.Y., Gopalsamy B., Abdul Rahim R., Manaf Ali A., Haji Lajis N. (2021). Anticancer Potential of Damnacanthal and Nordamnacanthal from Morinda elliptica Roots on T-lymphoblastic Leukemia Cells. Molecules.

[26] Hu X.M., Li Z.X., Lin R.H., Shan J.Q., Yu Q.W., Wang R.X., Liao L.S., Yan W.T., Wang Z., Shang L. (2021). Guidelines for Regulated Cell Death Assays: A Systematic Summary, A Categorical Comparison, A Prospective. Front. Cell Dev. Biol..

[27] Caruso S., Poon I.K.H. (2018). Apoptotic Cell-Derived Extracellular Vesicles: More than Just Debris. Front. Immunol..

[28] Doonan F., Cotter T.G. (2008). Morphological assessment of apoptosis. Methods.

[29] Xu X., Lai Y., Hua Z.C. (2019). Apoptosis and apoptotic body: Disease message and therapeutic target potentials. Biosci. Rep..

[30] Zhang Y., Sun R., Geng S., Shan Y., Li X., Fang W. (2019). Porcine Circovirus Type 2 Induces ORF3-Independent Mitochondrial Apoptosis via PERK Activation and Elevation of Cytosolic Calcium. J. Virol..

[31] Battistelli M., Falcieri E. (2020). Apoptotic Bodies: Particular Extracellular Vesicles Involved in Intercellular Communication. Biology.

[32] Kerr J.F.R., Wyllie A.H., Currie A.R. (1972). Apoptosis: A Basic Biological Phenomenon with Wideranging Implications in Tissue Kinetics. Br. J. Cancer.

[33] Vijayarathna S., Chen Y., Kanwar J.R., Sasidharan S. (2017). Standardized Polyalthia longifolia leaf extract (PLME) inhibits cell proliferation and promotes apoptosis: The anti-cancer study with various microscopy methods. Biomed. Pharm..

[34] Elmore S. (2007). Apoptosis: A review of programmed cell death. Toxicol. Pathol..

[35] Wong R.S. (2011). Apoptosis in cancer: From pathogenesis to treatment. J. Exp. Clin. Cancer Res..

[36] Aoki K., Parent A., Zhang J. (2000). Mechanism of damnacanthal-induced [Ca^2+^]i elevation in human dermal fibroblasts. Eur. J. Pharmacol..

[37] Berridge M.J., Irvine R.F. (1984). Inositol triphosphate, a novel second messenger in cellular signal transduction. Nature.

[38] Chakraborti T., Das S., Mondal M., Roychoudhury S., Chakraborti S. (1999). Oxidant, mitochondria and calcium: An overview. Cell. Signal.

[39] Takeyama N., Matsuo N., Tanaka T. (1993). Oxidative damage to mitochondria is mediated by the Ca2+-dependent inner-membrane permeability transition. Biochem. J..

[40] Siman R., Noszek J.C. (1988). Excitatory amino acids activate calpain I and induce structural protein breakdown in vivo. Neuron.

[41] Verity M.A. (1993). Mechanisms of phospholipase A2 activation and neuronal injury. Ann. N. Y. Acad. Sci..

[42] Granville D.J., Carthy C.M., Hunt D.W.C., McManus B.M. (1998). Apoptosis: Molecular aspects of cell death and disease. Lab. Investig..

[43] Li W., Shi X., Xu Y., Wan J., Wei S., Zhu R. (2017). Tamoxifen promotes apoptosis and inhibits invasion in estrogen-positive breast cancer MCF-7 cells. Mol. Med. Rep..

[44] Liu C.-Y., Hung M.-H., Wang D.-S., Chu P.-Y., Su J.-C., Teng T.-H., Huang C.T., Chao T.T., Wang C.Y., Shiau C.W. (2014). Tamoxifen induces apoptosis through cancerous inhibitor of protein phosphatase 2A-dependent phospho-Akt inactivation in estrogen receptor-negative human breast cancer cells. Breast Cancer Res..

[45] Pilco-Ferreto N., Calaf G.M. (2016). Influence of doxorubicin on apoptosis and oxidative stress in breast cancer cell lines. Int. J. Oncol..

[46] Matsumoto M., Nakajima W., Seike M., Gemma A., Tanaka N. (2016). Cisplatin-induced apoptosis in non-small-cell lung cancer cells is dependent on Bax- and Bak-induction pathway and synergistically activated by BH3-mimetic ABT-263 in p53 wild-type and mutant cells. Biochem. Biophys. Res. Commun..

[47] Ismail N.H., Ali A.M., Aimi N., Kitajima M., Takayama H., Lajis N.H. (1997). Anthraquinones from Morinda elliptica. Phytochemistry.

[48] Hirose Y. (1960). Syntheses of Damnacanthal, Damnacanthol, Norjuzunal and Norjuzunol, the Coloring Matters of *Damnacanthus* spp.. Chem. Pharm. Bull..

[49] Adnan N.E. (2018). Isolation and photophysical properties of Di- and Tri-substituted natural anthraquinones from Malaysian Morinda citrifolia. Sains Malays..

[50] Lillie R.D.H.J. (1977). Conn’s Biological Stains.

